# The short-term impacts of coronavirus quarantine in São Paulo: The health-economy trade-offs

**DOI:** 10.1371/journal.pone.0245011

**Published:** 2021-02-17

**Authors:** Alexandre Gori Maia, Leticia Marteleto, Cristina Guimarães Rodrigues, Luiz Gustavo Sereno

**Affiliations:** 1 Center for Applied Economics, Agriculture and the Environment, University of Campinas, Campinas, São Paulo, Brazil; 2 Population Research Center and Department of Sociology, University of Texas, Austin, TX, United States of America; 3 Institute of Economic Research Foundation, University of São Paulo, São Paulo, Brazil; Universidad Nacional Autonoma de Nicaragua Leon, NICARAGUA

## Abstract

We analyze the trade-offs between health and the economy during the period of social distancing in São Paulo, the state hardest hit by the COVID-19 pandemic in Brazil. We use longitudinal data with municipal-level information and check the robustness of our estimates to several sources of bias, including spatial dependence, reverse causality, and time-variant omitted variables. We use exogenous climate shocks as instruments for social distancing since people are more likely to stay home in wetter and colder periods. Our findings suggest that the health benefits of social distancing differ by levels of municipal development and may have vanished if the COVID-19 spread was not controlled in neighboring municipalities. In turn, we did not find evidence that municipalities with tougher social distancing performed worse economically. Our results also highlight that estimates that do not account for endogeneity may largely underestimate the benefits of social distancing on reducing the spread of COVID-19.

## Introduction

Nonpharmaceutical interventions (NPIs) are a critical recommendation in halting the spread of COVID-19 in the absence of an effective treatment or vaccine [1]. Social distancing is a key NPI, which aims to increase the distance between individuals and to reduce the frequency of social interactions through, for example, the closure of workplaces and schools, and the suspension of social activities, events and large gatherings [2]. While knowledge on the effects of social distancing in containing the pandemic is developed, a question arises on whether social distancing hurts the economy while helping health conditions.

Recent analyses on the health-economy trade-offs of social distancing suggest that the benefits of lives saved may exceed the economic costs of recession in the short-term [3]. While a premature lifting of social distancing measures may temporarily raise economic activity, subsequent deaths due to COVID-19 may hit economic growth in the long run [4]. Further analysis also suggests that a targeted shielding of the elderly and a combination of testing/tracing/isolating infected individuals are alternatives to uniform social distancing measures. In a study focusing on the U.S., twice as many lives could be saved if governments prioritized the allocation of resources to protect the most vulnerable rather than imposing measures on those who face significantly lower risk of death [5]. Examining both short- and long-term consequences, Pindyck shows that reducing the reproduction rate of infection—the average number of secondary cases per infectious case—reduces the number of deaths in the short-term [6]. However, it also extends the pandemic’s economic costs in the long-run since it increases the population still susceptible to new coronavirus infections and raises the possibility of a second wave.

While contributing to the understanding of the health-economy trade-offs of social distancing measures, these studies have mainly focused on developed countries and relied on projections of epidemiological models (SIR models). Developing countries face different trade-offs than developed nations in the fight against COVID-19 because they have large informal sectors, a younger population, and limited fiscal room for prolonged policies of social distancing. Another critical issue is that social distancing is unlikely to achieve the desired health benefits if lifted too soon [7], and, particularly in developing countries, NPIs have not been successful in effectively controlling the spread of COVID-19 in the short-term [8]. Further limiting current evidence, studies based on SIR models assume an unrealistic theoretical and standard infection rate for all population groups [6]. The reliability of SIR projections also depends mostly on the ability to calibrate the model to the observed data and confirm estimates with real behavioral patterns [9]. As data on infection and social distancing become available, studies based on observed data may provide more accurate patterns [10] and valuable variation to illustrate the reality in other parts of the world and between different social groups.

We use a rich longitudinal sample of observed municipal-level information to examine the short-term health-economy trade-offs of social distancing in the state of São Paulo (SP), Brazil. Brazil was the country with the second-largest number of reported cases and deaths due to COVID-19 in the early months of the pandemic [11]. By June 30, Brazil had 1.4 million confirmed cases of COVID-19 and 60,000 deaths with positive COVID-19 testing [12]. We focus on SP because it is Brazil’s hardest-hit state, provides excellent data quality, and presents a sizeable socioeconomic heterogeneity across municipalities, allowing us to make meaningful comparisons. SP also concentrates nearly one-third of Brazil’s economic activity [13]; if SP economic activity is affected, the entire country tends to be affected. SP is also an interesting case to examine because the state implemented a statewide social distancing policy in late March, though compliance with the policy varied across municipalities.

We evaluate the impacts of social distancing on health and economic indicators. The health indicators are the rate of confirmed cases of COVID-19 and the rate of deaths confirmed with COVID-19 testing (per 1,000 people). The economic indicators are the municipal tax collection revenue and the rate of net employment losses. Our empirical strategy combines instrumental variable estimators and spatial panel models to overcome methodological challenges common to policy evaluations [14, 15]. The first challenge is omitted variables: social distancing measures may be associated with unobservable determinants of health and economic indicators, such as social behavior. The second is reverse causality: the risk of contagion may also influence the decision to stay at home. The third challenge for this kind of analysis that we overcome in this study is spatial dependence; that is, the possibility that health and economic outcomes are also affected by neighboring municipalities’ outcomes.

Our findings show that estimates that do not account for these potential sources of endogeneity may be largely biased. We find robust evidence for the benefits of social distancing impacts on health indicators, which differed by municipalities’ levels of socioeconomic development. Our findings also highlight that the health benefits of social distancing may have vanished if the COVID-19 spread was not controlled in neighboring municipalities, i.e., measures of social distancing should be coordinated at the regional level. However, we do not find evidence that municipalities with above-average social distancing practices performed economically worse than municipalities with below-average social distancing.

## Background section: Social distancing policies and the healthcare system in the face of the COVID-19 pandemic

Brazil confirmed the first case of COVID-19 on February 25 in the municipality of São Paulo, the capital of SP state [2]. Four months later, at the end of June, the state had approximately 280 thousand confirmed cases (nearly 1/5 of all reported cases in Brazil) and about 15 thousand deaths with positive COVID-19 testing [12]. São Paulo city alone, with nearly 12 million people (nearly ¼ of the total state population), reported around 150 thousand cases and 7 thousand deaths by the end of June (nearly half of the cases and deaths in the state). COVID-19 cases were concentrated in poor neighborhoods, where people are packed in slums and have informal jobs, making social distancing extremely hard [16].

The lack of coordination among state and federal health agencies may have had detrimental impacts on the fight to contain the spread of COVID-19 [17]. The federal government downplayed the pandemic’s severity, and Brazil lost two health ministers in the first months of the crisis. As a result, the Ministry of Health faced difficulties implementing a contingency plan to handle the pandemic, such as measures to support the testing and diagnosis of COVID-19 and social distancing [18]. The lack of coordinated efforts culminated with governors acting mainly at the state level.

Brazil’s federalist system gives states further autonomy to implement state-level policies to contain public health emergencies [19]. The first policy adopted in SP was on March 13 and consisted of emergency social distancing measures, such as the suspension of in-person classes and events with more than 500 people [20]. On March 20, SP canceled all non-essential activities (e.g., religious events) and in-person governmental services, as well as closed state parks [21]. On March 22 the state issued further restrictions on public and private non-essential activities.

The state subsequently issued a plan to resume non-essential activities (*Plano SP*) that includes five stages [22]: maximum level of restriction for non-essential activities (red), control (orange), flexibility (yellow), partial opening (green), and usual controlled (blue). Each of the state’s 17 regions was classified according to a stage, following criteria such as hospital capacity and the evolution of confirmed COVID-19 cases. With the exception of four regions classified in stage yellow, all remaining regions were classified as red or orange within the whole period we examine. Municipalities had some autonomy to change state restrictions provided that they presented technical evidence to support their decision to the state government. It is important to note that SP never implemented a full lockdown policy; that is, neither the state nor municipal governments issue mandatory measures restricting the circulation of people on specific days of the week or times of the day.

SP also implemented further protocols such as patient screening and creating field hospitals [23]. However, the protocol lacked a policy of mass testing. Early on in the pandemic, only those who required hospitalization were tested, in addition to healthcare providers, the elderly in nursing homes, and the incarcerated population. On May 18, SP adopted a broader protocol for testing that included those in contact with people who tested positive for COVID-19 and presented mild asymptomatic cases: nearly 500 thousand people were tested in May [24]. Testing became more widely available in June—nearly 900 thousand people were tested. We control for time differences in the number of COVID-19 testing using fixed effects (explained in more detail below).

SP is the wealthiest state in Brazil and has a relatively strong health system, which helped the state coping with the pandemic [25]. While Brazil provides a universal and public health system free of charge (SUS, *Sistema Único de Saúde*), the coverage and quality differ broadly across states [26]. For example, SP had 19 ICU beds and 39 respirators by 100,000 inhabitants designated to treat COVID-19 patients, while the federal government recommended ten beds and two respirators by 100,000 inhabitants [27]. The private health system also covers a large share of SP’s population, nearly 40% [28], which helped avoid the state’s public healthcare system collapse.

## Data and empirical strategy

### Data and variables of interest

Our indicator of social distancing (variable *I*) is provided by the *Fundação Sistema Estadual de Análise de Dados* (SEADE), from the SP state [12]. *I* is based on the location obtained by cell phone antennas, which defines a reference point where each mobile phone was placed between 10 pm and 2 am. The mobile is considered “out of isolation” or “in distance” during the day if it moved from this reference point to another point situated beyond a minimum distance. The minimum distance depends on the municipality, but it is usually around 200 meters. *I* represents the proportion of mobiles in isolation (did not move beyond 200 meters) in each municipality. This indicator is similar to those used in prior studies in Brazil and abroad as a proxy for social distancing [29–31]. Variation in *I* may reflect both differences in the municipal measures of social distancing and compliance to the measures, i.e., how specific policies translated into effects.

SEADE provides daily values of *I* for 104 municipalities in the state of SP (Map 1) starting on March 5, 2020. The data are available for municipalities with cell phone coverage by the four leading cell phone companies in São Paulo; combined, these four companies have 97% of the state coverage [32]. The data is processed directly by the phone companies and made available by SEADE without any personal identification, following the Brazilian General Law on Protection of Personal Data [33].

**Fig 1:**
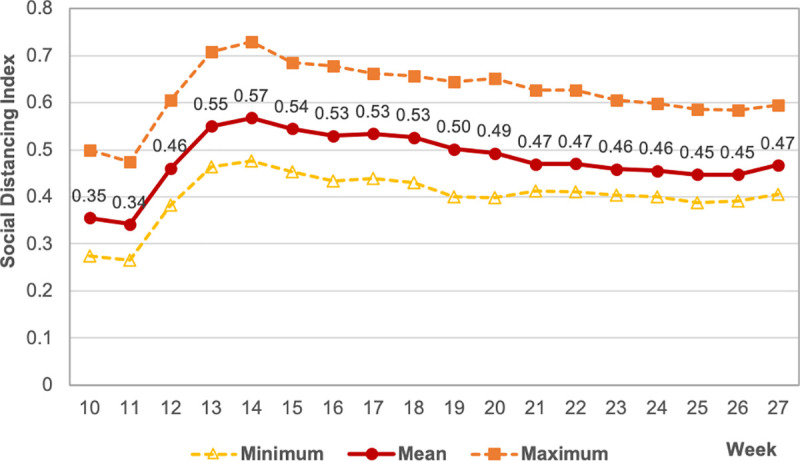
Weekly municipal social distancing index (*I*), sampled municipalities São Paulo state, weeks 10 to 27, 2020.

We initially computed the municipal weekly averages between week 10 (between March 1–7) and week 27 (June 28—July 4) of 2020. Our first sample contains a balanced panel with 104 municipalities (*i* = 1..104) and 18 weeks (*t* = 10..27). In the first two weeks of March, when social distancing measures were not yet in place in SP, *I* was around 35% (week 10) and 34% (week 11) (Fig 1). Social distancing reached a peak in the first two weeks of the state’s official quarantine: average *I* of 55% in week 13 and 57% in week 14. Since then, social distancing has lost pace in SP, falling to 47% in week 27.

We matched the SEADE sample of social distancing indices with weekly data for two dependent variables at the municipal level: confirmed cases of COVID-19 per 1,000 people; deaths with positive COVID-19 testing per 1,000 people. Cases and deaths are those with a positive test result for COVID-19, independent of hospitalization, and municipalities refer to the place of residence [34]. We computed the ratio between the new cases (or deaths) per week and the total population in each municipality using the estimates of municipal population size provided by SEADE, which are assumed to be constant in the period [35]. We restricted the analysis to weeks 10 to 27 to match the data for cases and deaths with positive COVID-19 testing with the available data for *I*.

**Fig 2:**
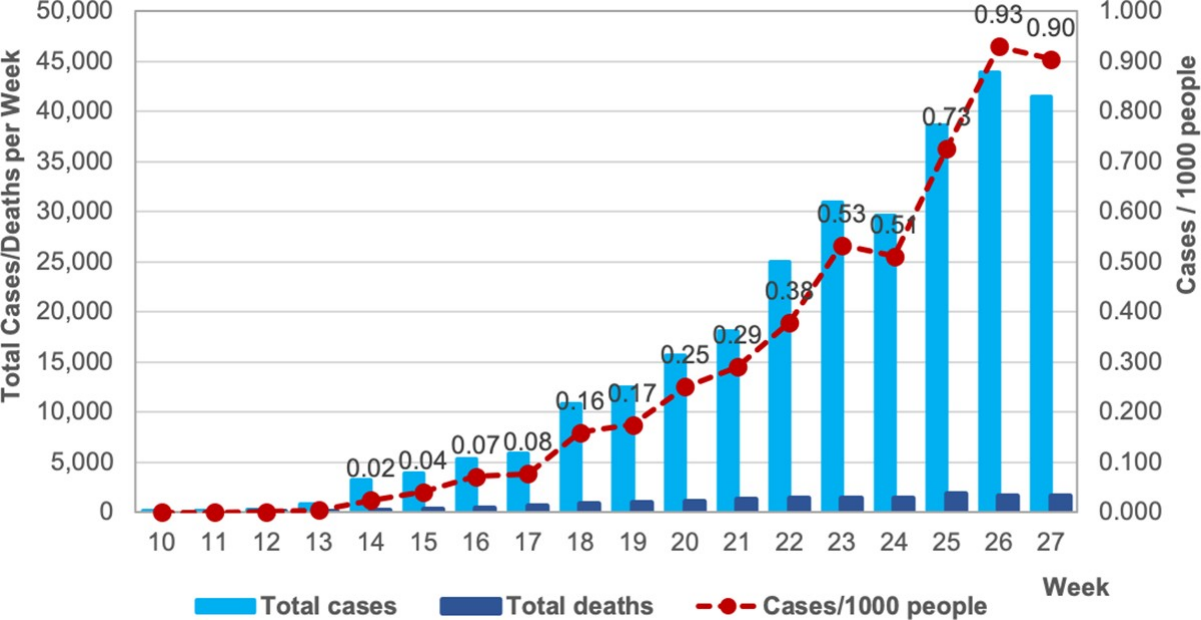
New confirmed cases, deaths and rates of COVID-19 infections, sampled municipalities in São Paulo state, weeks 10 to 27, 2020.

Essential for this research, despite this restriction, our analytical sample of municipalities contains the hotspots of COVID-19 cases in SP: 91% of the total cases in the state up to week 27. The number of confirmed new cases of COVID-19 increased at a rate of 67% per week between weeks 10 and 27 (Fig 2), going from 11 to 41,410 new cases per week. In week 26, the rate of new cases reached 0.93 per 1,000 people. The first confirmed death attributed to COVID-19 occurred in week 12. Since then, the number of deaths with positive COVID-19 testing increased from 9 in week 12 to 1,598 in week 27. To provide a sense of how this compares to total mortality, the total number of deaths with positive COVID-19 testing represented nearly 8% of the total deaths in the state of SP in April (2,239 of 26,425) and 23% in June (7,148 of 30,886) [36].

We also examine two economic dependent variables: the net change in employment by 100 employed people (net employment rate) and the log Tax Revenue on Commerce and Services (ICMS, or *Imposto sobre Circulação de Mercadorias e Serviços*). The net change in employment is the difference between gross job gains and gross job losses in each municipality and month. This information is provided by the General Registry of Employees and Unemployed (CAGED, *Cadastro Geral de Empregados e Desempregados*), from the Ministry of Labor and Employment, and refer exclusively to formal jobs. We computed the ratio (by 100 people) between the net change in employment and the total number of formal employees in December 2018 using the information provided by the Annual Report of Social Information (RAIS, *Relação Annual de Informações Sociais*), also from the Ministry of Labor and Employment. The ICMS is the main revenue tax levied by the state of São Paulo (84% of the total tax collection) and applies to the movement of goods, transportation and telecommunications services. We used monthly municipal-level information for the tax collection from the São Paulo State’s Department of Finance and Planning [37].

**Fig 3:**
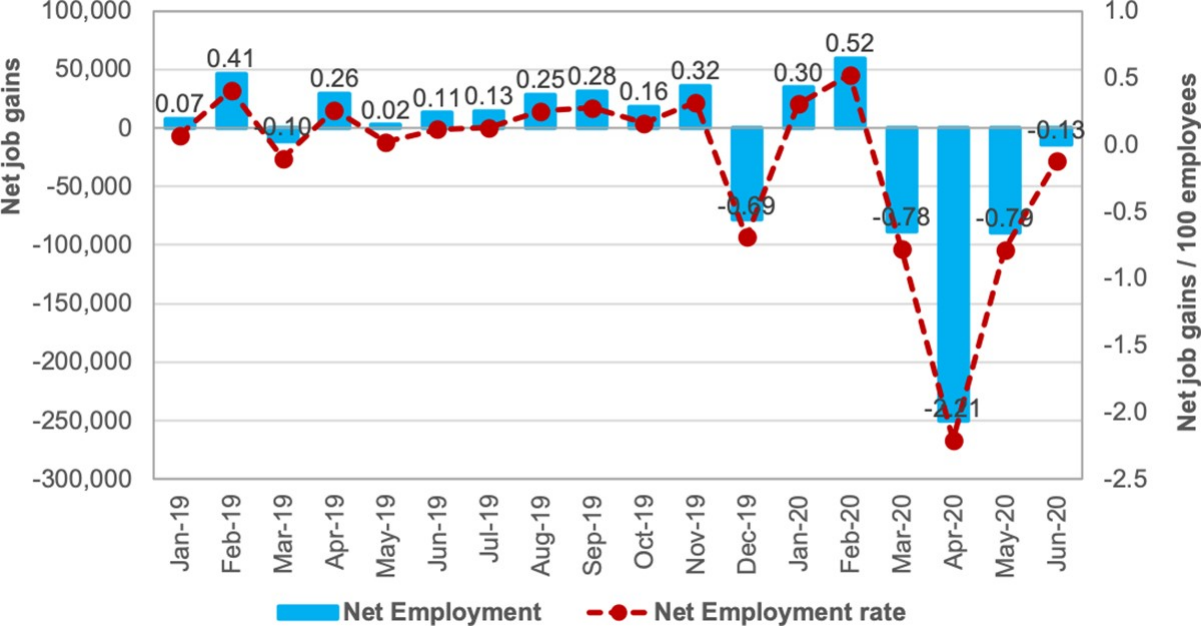
Net change in employment, sampled municipalities in São Paulo state, January 2019 to June 2020.

**Fig 4:**
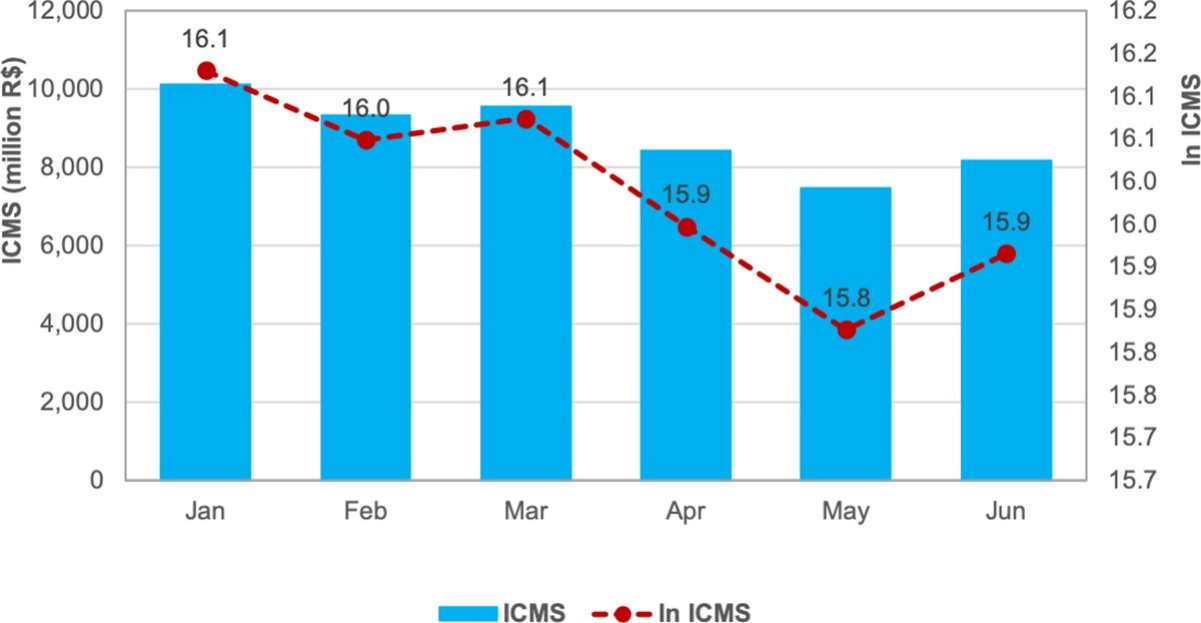
Tax collection of ICMS, sampled municipalities in São Paulo state, January to June 2020.

We matched the monthly net employment rate and tax data with the monthly averages of *I*. This sample contains a panel with 104 municipalities across four months, March, April, May, and June (*t* = 3..6). These municipalities concentrate the largest share of the SP economy: nearly 85% of the state’s formal employment and nearly 90% of the states’ tax revenue. Fig 3 shows that these municipalities lost 440,852 formal jobs during the first four months of official quarantine, between March and June 2020 (Fig 3). This net job loss is almost twice as large as the yearly net job gains between January 2019 and February 2020 (230,000 jobs) and represents 3.9% of the state’s formally employed population in December 2018. The tax collection is more elastic to the economic crisis and fell by 22% between March and May 2020: from 9.6 to 7.5 billion Brazilian Reais (Fig 4). The tax collection revenue rebounded in June, growing by 9.3% in relation to May.

We tested how moderator factors may affect the causal relationship between social distancing and the health and economy. The moderator factors are time-invariant variables that are interacted with *I*. From the Demographic Census 2010, of the Brazilian Census Bureau (IBGE) [38], we obtained the following moderator factors at the municipality level that may be related to the vulnerability to COVID-19: (i) elderly population, share of households headed by individuals age 65 or older; (ii) poverty rate, the share of the population living on less than R$ 255 per month (nearly U$ 50); (iii) no water access, the share of the population without access to piped water in the household; (iv) household crowding, share of the population living in households with more than two members per bedroom.

Next, we used data from RAIS 2018 to check how social distancing may have differently affected the economic dynamics. Our moderator factors for the impacts of social distancing on net employment rate and log tax are the shares of formal workers in each of SP’s leading economic sectors. These sectors are agriculture, manufacturing, construction, retail trade, food and housing, and social services (public sector, education, and health services).

**Fig 5:**
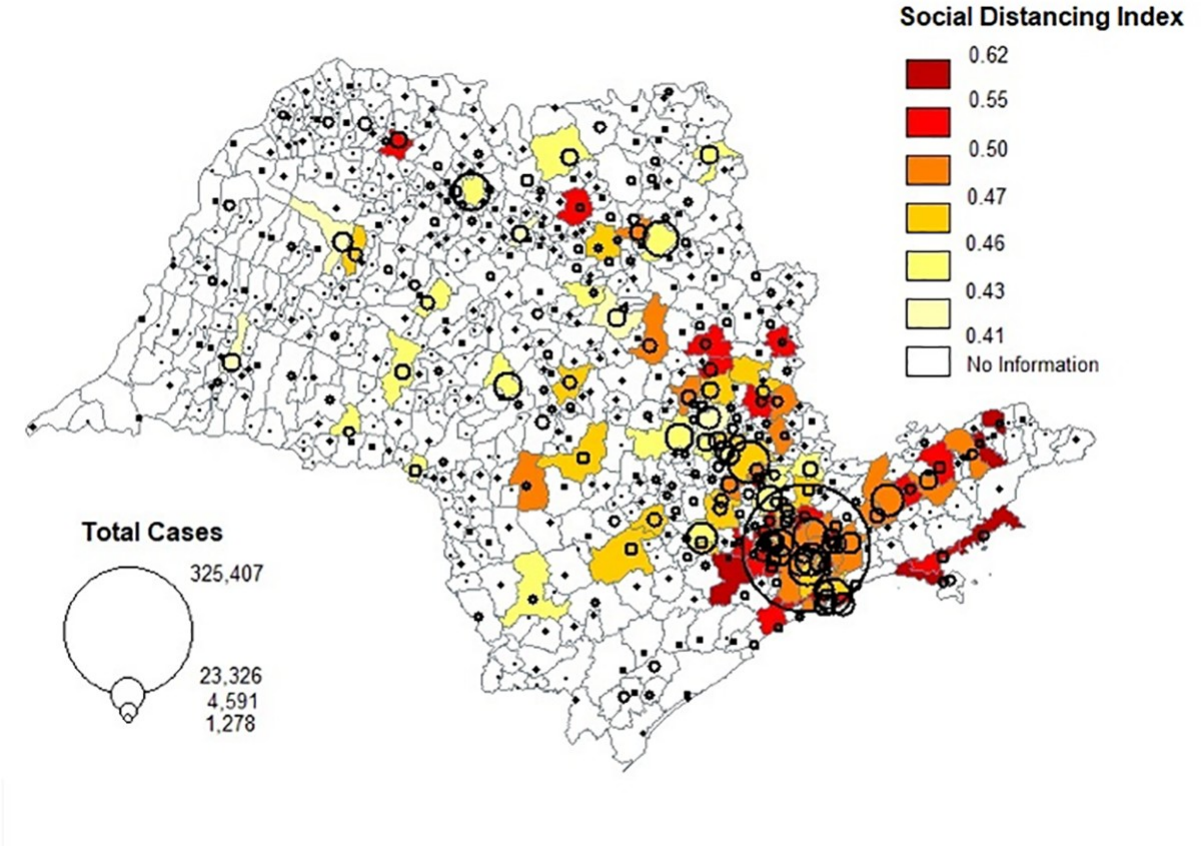
Total number of COVID-19 cases (circles) and average social distancing index (colors) per municipality, São Paulo, 2020.

Table 1 presents the municipal averages of our variables of interest for our analytical sample of municipalities. Fig 5 shows the spatial distribution of the total number of confirmed COVID-19 cases (proportional circles) and the average *I* across municipalities with non-missing values (groups of colors).

**Table 1:** Mean and standard deviation (S.D.) of variables of interest, sampled municipalities in São Paulo state, 2020.

Variable	Definition	Mean	S.D.
*Interest variable*			
Social distancing	Share of mobiles which did not move	0.479	0.072
*Dependent Variables*			
Rate of new cases	New cases of COVID-19 per 1,000 people	0.282	0.455
Rate of new deaths	New deaths with positive Covid-19 testing per 1,000 people	0.014	0.022
Net employment rate	Difference between job gains and job losses by 100 formal employees	−0.952	1.430
ICMS ~ Tax Revenue	Collection of tax on commerce and services (1,000 Reais)	80,871	297,599
*Instrumental variables*			
Temperature	Average temperature in Celsius	21.551	1.989
Precipitation	Total precipitation in decimeters	0.069	0.125
*Moderators for health*			
Poverty rate	Share of people living on less than R$ 255 per month (≈50 dollars)	0.142	0.053
No water access	Share of people without household piped water	0.075	0.078
Household crowding	Share of people living in households with an average of more than 2 members per bedroom	0.193	0.072
Elderly population	Share of households headed by individuals ages 65 or older	0.141	0.035
*Moderators for economy*			
Agriculture	Share of formal employees in agriculture	0.026	0.050
Manufacturing	Share of formal employees in manufacturing	0.230	0.114
Construction	Share of formal employees in construction and real state	0.045	0.028
Retail trade	Share of formal employees in wholesale and retail trade	0.225	0.046
Hotel and restaurant	Share of formal employees in hotels and restaurants	0.045	0.026
Social Service	Share of formal employees in the public, education and health services	0.201	0.054
Others	Share of formal employees in other sectors	0.228	0.089

Source: SEADE, São Paulo State’s Department of Finance, INMET, IBGE, and Ministry of Labor and Employment.

Table 1 shows that our sample represents some of the most developed and densely populated municipalities in the country. For example, the poverty rate was 14.2% (16.6% in other municipalities of São Paulo and 38.5% in the rest of the country), and household crowding affected 19.3% of the population (13.6% in other municipalities of São Paulo and 16.9% in other Brazilian municipalities). The economy is characterized by manufacturing (23% of the formal employees, against 20.5% in the rest of the state and 12.4% in the rest of Brazil) and the retail trade sector (22.5% of the formal employees, against 16.1% in the rest of the state and 15.5% in the rest of Brazil).

### Empirical strategy

We combined models for *I*-health indicators and *I*-economic indicators to analyze the health-economy trade-offs of social distancing, i.e., we fitted one model for each dependent variable. The benchmark model is represented by Eq (1):

$$Y_{it}=\rho w_{i}Y_{t}+I_{it}\delta +c_{i}+d_{t}+\varepsilon_{it}$$ (1)

Where *Y*_*it*_ is the outcome of interest (rate of confirmed cases of COVID-19, rate of deaths with positive COVID-19 testing, net employment rate, or log tax) and **I**_*it*_ is the vector of contemporaneous and lag values of *I* (variables *I*_*t*−*k*_, where *k* = 0,1 or 2) at municipality *i* and period *t*. Our main interest is the vector of coefficients **δ**, representing the net impacts of social distancing on the outcome *Y*. Time-invariant characteristics that vary between municipalities (such as the healthcare system and socioeconomic development) are represented by *c* and are controlled using fixed effects. Time-varying characteristics that are constant between municipalities (number of COVID-19 tests, seasonality in deaths, and economic activity, for example) are represented by *d* and are controlled by using binary variables for the periods. The idiosyncratic error *ε* represents the random factor between municipalities and time that is not controlled by the model.

Eq (1) also controls for spatial dependence. Regional dynamics may jointly influence health and socioeconomic conditions, i.e., events in the municipality *i* may be influenced by events originating in spatially nearest municipalities. For example, COVID-19 cases in some municipalities may spread to nearby municipalities and influence both the number of cases and policies of social distancing in other municipalities. The variable **w**_*i*_**Y**_*t*_ controls for neighborhood effects on municipality *i*. The vector **w**_***i***_ is the inverse-distance spatial-weighting vector, which contains the inverse of the distance between municipality *i* and the five nearest municipalities in the sample. The distances are normalized in order to have a sum equal to 1. The vector **Y**_*t*_ contains the values of *Y* for all municipalities at week *t*, and **w**_*i*_**Y**_*t*_ is the average outcome in the neighbor municipality of *i* at period *t*. The parameter *ρ* is the coefficient spatial autocorrelation of *Y*, which may be, for example, a proxy for the rate of spatial spread of COVID-19.

Eq (1) controls for most confounders that might be jointly related to the dependent and independent variables. However, we may still have reasons to believe that *I* is endogenous due to both reverse causality and omitted variable bias. Reverse causality may occur if policies of social distancing are a response to new cases of COVID-19. In turn, omitted variable bias may arise due to time-varying unobservable factors that differ between municipalities, such as social acceptance of policies to contain the spread of COVID-19. In this case, the fixed-effects estimators used in Eq (1) may be biased and inconsistent.

We used the control function (CF) method to control potential sources of endogeneity bias [39]. The CF method includes a proxy for the correlation between *I* and the idiosyncratic error term *ε* in Eq (1). The estimates of the CF approach are obtained in two stages. In the first stage, we use fixed effects estimators to fit a model for *I* as a function of binary variables for the time periods plus additional exogenous instrumental variables (IVs). In the second stage, we fit Eq (1) adding the residuals from the first stage. The main advantage of the CF approach in relation to traditional two-stage-least-squares (2SLS) estimators is that the CF provides consistent estimates in the context of non-linear models, while the 2SLS estimates may be inconsistent [40].

The consistency of the CF estimators relies on three main assumptions. The first is the relevance assumption, which assumes that the IVs have a causal effect on COVID-19 cases and deaths. The second assumption is the exclusion restriction, which assumes that the IVs have no direct impact on COVID-19 cases and deaths, only indirectly through the *I*. The third assumption is the ignorability, which assumes that the IVs are not related to unobservable determinants of COVID-19 cases and deaths. We use two weather variables as IVs (Table 1): weekly total precipitation and weekly mean temperature. The weather variables satisfy the relevance assumption since they are strongly related to *I*. S1 Appendix shows the estimates for the first stage (equation for *I*), highlighting that social distancing increases with high precipitation and decreases with high temperature. The weather variables may also satisfy the second (exclusion restriction) and third (ignorability) assumptions. Studies have shown that, after controlling for sociodemographic factors, weather alone had a negligible effect on the spread of COVID-19 across the world [41]. In other words, the correlation between weather and COVID-19 found in some studies [42] may arise because of the influence of weather on people’s behavior, rather than on the virus itself. The results of our overidentification tests also reinforce the exclusion restriction and ignorability assumptions. The Hansen statistics (Tables 2 and 3, to be presented below) are insignificant at 5%, i.e., mean temperature and total precipitation are exogenous (unrelated to the idiosyncratic errors *ε*).

**Table 2:** QML and GMM estimates for the dependent variables rate of new cases and rate of new deaths with positive COVID-19 testing, controlling for spatial dependence, sampled municipalities in São Paulo state, weeks 10 to 27 of 2020.

Variable	Cases per 1,000								Deaths per 1,000							
	Model 1				Model 2				Model 1				Model 2			
	QML		GMM		QML		GMM		QML		GMM		QML		GMM	
*I*_*t*_	−4.901		0.002		−5.024		−1.570		−0.617	**	0.000		−0.658	***	−0.034	
	(5.602)		(0.004)		(3.929)		(5.576)		(0.198)		(0.000)		(0.177)		(0.035)	
*I*_*t*−1_					−4.007		1.732						−0.141		0.000	
					(4.188)		(5.230)						(0.182)		(0.053)	
*I*_*t*−2_					2.241	***	−0.479						0.159	***	0.027	
					(0.442)		(0.598)						(0.033)		(0.037)	
**wY**	0.486	***	0.812	***	0.464	***	0.994	***	0.394	***	0.992	***	0.330	***	0.910	***
	(0.069)		(0.129)		(0.069)		(0.229)		(0.41)		(0.109)		(0.043)		(0.120)	
Municipalities	104		104		104		104		104		104		104		104	
Weeks	18		18		16		16		18		18		16		16	
*R*^2^ (within)	0.598		0.670		0.605		0.644		0.440		0.504		0.442		0.489	
Hansen test (*p*-value)	0.538		0.253		0.520		0.082		0.882		0.946		0.221		0.346	

*** p<0.001; ** p<0.01; * p<0.05, + p<0.10. Robust estimates for the standard errors between parentheses.

**Table 3:** QML and GMM estimates for the dependent variables net employment rate and log tax revenue, controlling for spatial dependence, sampled municipalities in São Paulo state, March to June 2020.

Variable	Net Employment Rate				Log Tax Revenue			
	Model 1		Model 2		Model 1		Model 2	
*I*_*t*_	19.986		26.801	^+^	1.974		−0.231	
	(13.184)		(15.160)		(2.137)		(3.398)	
*I*_*t*−1_			4.880				4.720	^+^
			(10.023)				(2.643)	
**wY**	0.013		0.056		0.079		0.080	
	(0.066)		(0.068)		(0.060)		(0.071)	
Municipalities	104		104		104		104	
Months	4		3		4		3	
*R*^2^ (within)	0.389		0.615		0.287		0.226	
Hansen test (*p*-value)	0.240		0.280		0.731		0.800	

*** p<0.001; ** p<0.01; * p<0.05, + p<0.10. Robust estimates for the standard errors between parentheses.

We estimated Eq (1) using quasi–maximum likelihood estimators (QML) with fixed-effects [43]. This strategy fits Eq (1) adding the first stage residuals (equation for *I*) as an additional regressor for *Y*. We also checked the robustness of the QML estimates compared to the GMM estimates based on Baltagi et al. [44]. The GMM estimators are able to jointly account for the endogeneity of **wY** and *I* without using the residuals of the first stage. We use the two-step system GMM estimators, which include lagged levels and lagged differences of the regressors as instruments for the endogenous variables [45, 46]. We also included the weather variables (mean temperature and total precipitation) as exogenous instruments for **wY** and *I*. We did not use GMM when analyzing the monthly series for economic activity (net employment and ICMS) since the consistency of the GMM estimators also relies on a large number of periods.

## Results

### The impacts of social distancing on the spread of COVID-19

Table 2 shows the QML and GMM estimates for two dependent variables—the rate of confirmed cases and deaths with positive Covid-19 testing (Eq 1). QML estimates are from Stata’s *xsmle* command [43] and GMM estimates are from Stata’s *xtabond2* command [47]. Model 1 measures the contemporaneous impacts of social distancing (*I*_*t*_) while model 2 measures the lagged effects (the impacts of social distancing implemented at weeks *t*−1 and *t*−2, i.e., *I*_*t*−1_ and *I*_*t*−2_). Model 1 assumes that *I*_*t*_ is endogenous, and model 2 assumes that both *I*_*t*_ and *I*_*t*−1_ are endogenous, while *I*_*t*−2_ is assumed exogenous. The IVs in the QML are the weather variables, and the instruments in the GMM are both the weather variables and the lagged levels and differences of the regressors. The overidentification tests (Hansen’s p-value) indicate that the IVs for the endogenous regressors are valid (exogenous) in all models.

The direction and significance of the estimates tend to be similar for both dependent variables, suggesting the robustness of our estimates to the health indicator’s choice. The results also indicate that both variables present high levels of spatial dependence. The estimates of the coefficient of spatial autocorrelations for the rate of confirmed cases of COVID-19 range between 0.486 and 0.994, and, for the rate of deaths with positive COVID-19 testing, range between 0.394 and 0.992. These high coefficients indicate that the pandemic spread rapidly across neighboring municipalities.

The estimates in Table 2 provide moderate evidence that municipalities with higher levels of social distancing performed better when controlling for confirmed cases and deaths with positive COVID-19 testing. The QML estimates for the variable *I*_*t*_ are significant for the models of deaths with positive COVID-19 testing, while the GMM are mostly insignificant for both dependent variables. The QML estimates indicate that the weekly rate of deaths with positive COVID-19 testing per 1,000 people reduced by nearly 0.006 for each 1 percentage point increase in social distancing. If we consider the total population in our sample of municipalities (35.9 million people), the QML estimate suggests that nearly 215 lives (0.006×35.9 million/1,000) might have been saved per week for each 1 percentage point increase in social distancing. However, early measures of social distancing may have had detrimental impacts on the spread of COVID-19. For example, the QLM estimates for the variable *I*_*t*−2_ in the model for the rate of deaths with positive COVID-19 testing is 0.159. This estimate suggests that the number of deaths with positive COVID-19 testing per 1,000 people at week *t* may have increased by 0.00159 for each 1 percentage point increase in social distancing that took place two weeks earlier.

Besides endogeneity, the QML and GMM estimates also control for the COVID-19 dynamics (**wY**) in neighbor municipalities. In other words, we first assumed that the pandemic remained constant in neighboring municipalities. However, the pandemic may have also been controlled in the neighborhood due to social distancing in other municipalities. We checked the robustness of our results to the choice of the estimation strategy. S2 Appendix shows the fixed effect estimates using ordinary least square (OLS) and CF without control for spatial dependence, i.e., relaxing the hypothesis of constant cases and deaths in neighbor municipalities. OLS and CF estimates are from the *xtreg* command from Stata.

The OLS estimates do not control for endogeneity of *I* and seem to largely underestimate the impact of social distancing on reducing the spread of COVID-19. Most OLS estimates for *I*_*t*_ are positive and significant at 1%. In turn, the CF estimates for *I*_*t*_ are negative and significant at 5% in all models. The CF estimates in S2 Appendix are also larger in magnitude than those in Table 2. In other words, the level of social distancing at one municipality (*I*) seems to be strongly correlated to the rate of confirmed cases and deaths with positive COVID-19 testing in neighbor municipalities (**wY**). As a result, the impacts of social distancing on the rate of new cases and deaths reasonably decrease if we assume constant the cases and deaths in neighbor municipalities (control for **wY**).

**Fig 6:**
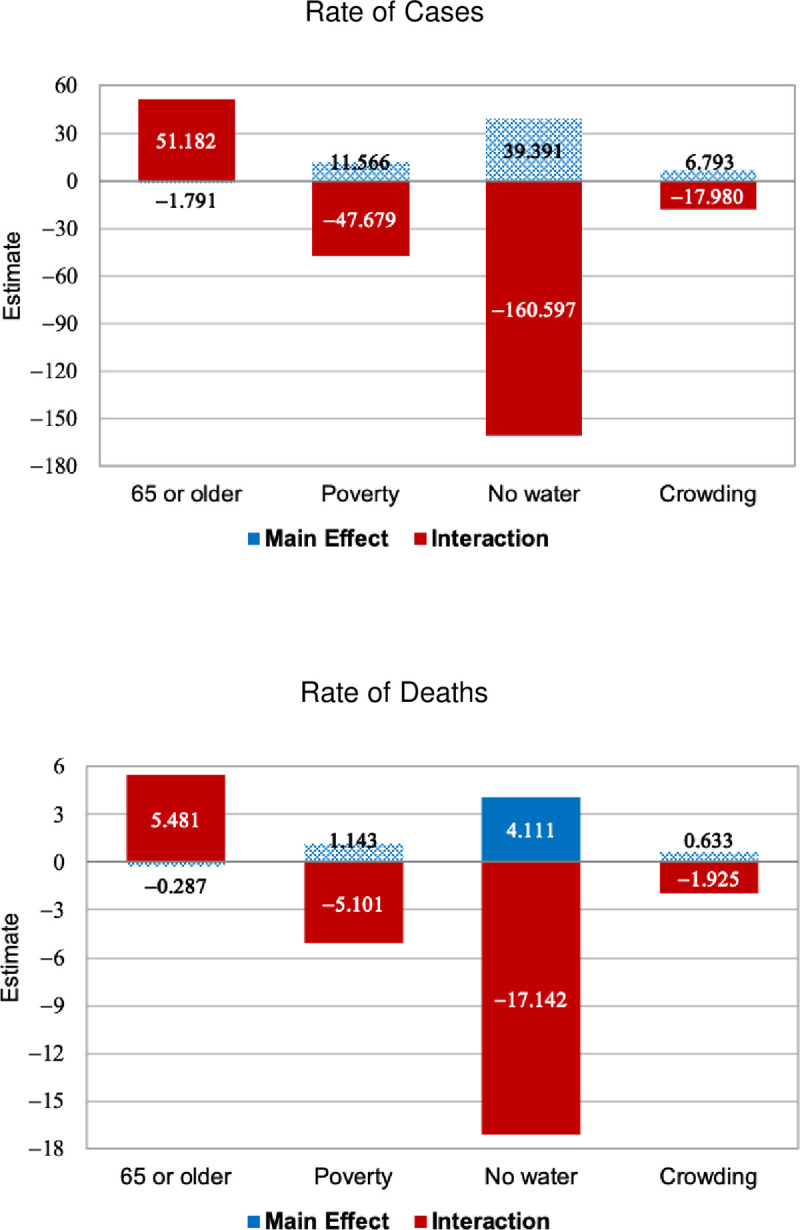
QML estimates for interactions between moderator factors and *I*, dependent variables: Rate of new cases and rate of new deaths with positive Covid-19 testing, sampled municipalities in São Paulo state, weeks 10 to 27 of 2020.

We next examined how the impact of social distancing on the spread of COVID-19 may have differed according to values of four moderator factors: elderly population, poverty rate, access to piped water, and household crowding. We fitted a model for each moderator (*M*), adding the interaction variable (*I*×*M*) in Eq (1). We assume that both variables (*I* and *I*×*M*) are endogenous and the IVs are the weather variables. Fig 6 summarizes the QML estimates for the main effect (the coefficient for *I*) and the interaction effect (the coefficient for *I*×*M*). The solid bars represent estimates that are significant at 5%. S4 Appendix shows the whole set of estimates and goodness of fit indicators.

The estimates for the interaction effects are significant at 5% in all models. The results also tend to be proportionally similar in direction and magnitude for both dependent variables. Further results show that social distancing was more effective in reducing the spread of cases and deaths with positive COVID-19 testing in the most vulnerable municipalities, i.e., municipalities with the largest share of poor households, crowded households, and those lacking access to piped water. For example, in municipalities with 60% of the households without access to piped water (the maximum value of our sample), new cases per week may have been reduced by nearly 0.57 per 1,000 people (39.391+0.60×(−160.597) = −56.967) for each one percentage point increase in *I*, and new deaths per week may have been reduced by nearly 0.06 per 1,000 people (6.793+0.60×(−17.980) = −6.172). In turn, social distancing showed detrimental impacts on municipalities with the largest shares of the elderly population. For example, the net impact of *I*_*t*_ on the cases per 1,000 increased by 0.51 point for each 1 percentage point increase in the share of households headed by individuals age 65 or older.

### The economic impacts of social distancing

Table 3 shows the estimates for the dependent variables net employment rate and log tax revenue. Model 1 measures the contemporaneous impacts of social distancing (month *t*), and model 2 measure the lagged effects (month *t*−1). Model 2 assumes that both *I*_*t*_ and *I*_*t*−1_ are endogenous. The overidentification tests (Hansen’s p-value) indicate that we have valid exogenous instruments for the endogenous regressors in all models. We were not able to apply GMM estimators due to the limited time series period. S3 Appendix presents the estimates without controlling for endogeneity (OLS) and spatial dependence (CF).

Our results do not show strong evidence that social distancing may have had significant short- (month *t*) or middle-term (month *t*−1) impacts on the local net employment rate or tax revenue. In other words, municipalities with higher social distancing levels did not necessarily perform worse economically than municipalities with lower social distancing levels. The estimates for models 1 and 2 are insignificant at 5%. Neither of these dependent variables shows significant patterns of spatial dependence, i.e., neither the rate of new job gains nor the log tax revenue in a municipality were affected by the dynamics in the neighboring municipalities, at least in the period we examine.

**Fig 7:**
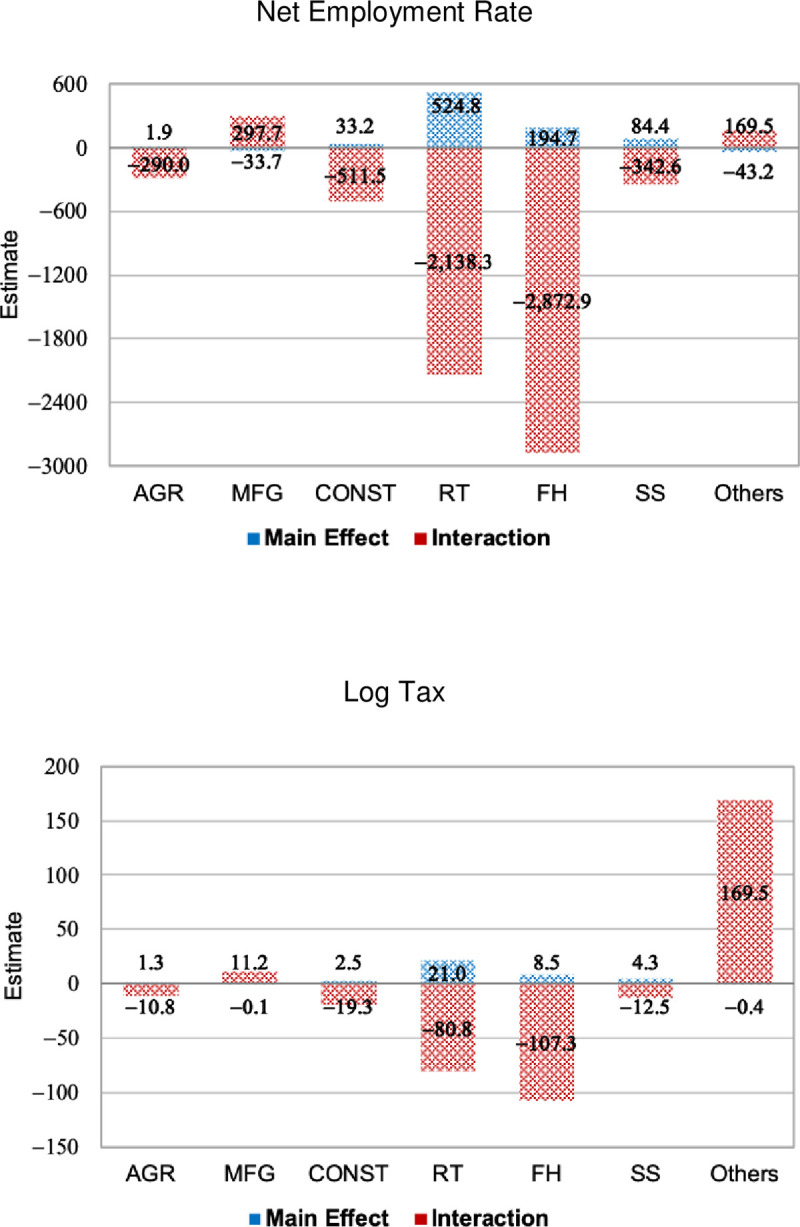
QML estimates for interactions between moderator factors and *I*, dependent variables: Net employment rate and log tax revenue, sampled municipalities in São Paulo state, March to June 2020.

We also checked whether the impact of social distancing on the economy might have differed according to the industry sector prevailing in each municipality, a way to consider sectoral heterogeneity across municipalities. We fitted seven models, each interacting the variable *I* with the share of formal employees (moderator *M*) in the municipality’s primary industry sector. We assumed that both *I* and *I*×*M* are endogenous and that the IVs are the weather variables. Fig 7 summarizes the QML estimates for the main and the interaction effects. S5 (the net employment rate) and S6 (log tax revenue) Appendices show the whole set of estimates and the measures of goodness of fit. The estimates are proportionally similar in direction and magnitude for both dependent variables. However, all estimates are insignificant at 5% (which are represented by dashed bars), suggesting that social distancing at the municipal level did not have significant net impacts on the municipal economy, regardless of the economic structure prevailing in the municipality.

## Discussion

In this study, we evaluated the health-economy trade-offs of social distancing in SP, the state with the largest number of confirmed COVID-19 cases in Brazil. SP is Brazil’s richest and most populous state, with a large diversity of socioeconomic structures that allow us to evaluate the heterogeneity of the health and economy trade-offs. Our results bring both applied and theoretical contributions to the growing literature on the socioeconomic and health impacts of the COVID-19 pandemic.

This study’s first contribution is to empirically show that social distancing did have significant benefits for controlling the spread of COVID-19. Our results also indicate that early measures to contain the spread of COVID-19 may have had detrimental impacts in the middle term. Both new cases and deaths with positive COVID-19 testing increased slightly due to the increases in social distancing that took place two weeks earlier.

We speculate that the success of social distancing in the short run may have generated an unanticipated *behavioral rebound effect* in the middle run; people may have felt more confident and relaxed recommendations to prevent the spread of COVID-19, such as social distancing, wearing masks, and handwashing. On the other hand, reducing the number of cases in the short term may extend the pandemic’s duration, increasing the population that will still be susceptible to infection in the middle- and long-terms [5]. Nonetheless, these results should be further investigated because the estimates are not robust among all empirical strategies.

Overall, the short-term health benefits of social distancing largely offset the detrimental impacts in the middle-run. In general, nearly 215 lives would have been saved for each 1 percentage point increase in the social distancing index in our analytical sample. The impacts of social distancing on the spread of COVID-19 also vary according to the municipality’s socioeconomic development level. People living in localities with poor infrastructures, such as lacking access to piped water and sanitation, face considerable difficulties in implementing social distancing and maintaining the high levels of personal hygiene required to prevent and contain the spread of COVID-19. Importantly, our findings suggest that it is precisely in most vulnerable municipalities that social distancing seems instrumental in containing the pandemic’s spread, i.e., social distancing was more effective in the poorest localities.

Another main contribution of this paper is that, although social distancing initially hit hard by cutting the number of formal jobs in SP, short-term variations in social distancing at the municipal level had insignificant impacts on the local number of formal jobs or tax revenue. In other words, municipalities with stricter social distancing measures did not necessarily perform worse economically than those with more relaxed social distancing measures. These results reinforce the idea that the COVID-19 pandemic is the main exogenous shock on the economy per se, rather than political decisions to extend or relax social distancing [10].

A third significant contribution of this study is that estimates of social distancing impacts that do not consider endogeneity may be largely biased. Social behavior and business restrictions may change as a response to the risk of infection, i.e., social distancing, COVID-19 spread, and the economy may be simultaneously determined. We used exogenous shocks of temperature and precipitation as instruments for social distancing. The population in SP showed to be more prone to stay home in the coldest and wettest periods.

Finally, a fourth finding suggests that the efforts to increase social distancing and curb the spread of COVID-19 within municipal borders may have vanished when similar measures were not adopted in neighboring municipalities. The spread of COVID-19 was strongly influenced by cases and deaths with positive COVID-19 testing in neighbor municipalities. This result suggests that measures to contain the spread of COVID-19 are not restricted to municipal borders, in a way that requires that public policies cross municipal boundaries. In other words, a regionally coordinated response might be the most appropriate measure to control the spread of COVID-19.

Combined, findings from this study suggest essential policy recommendations and critical limitations of the measures adopted in Brazil. First, social distancing measures should be coordinated at the regional level for higher effectiveness since there is a strong correlation between the pandemic spreading at the local and regional levels. Further, the lack of coordination between federal and state policies was an important limitation of the efforts to contain the COVID-19 pandemic in Brazil. More coordinated policies of social distancing, such as the regional plan to gradually re-open non-essential activities in SP (*Plano SP*), might have avoided the worst of the epidemic in Brazil. A broad consensus between the federal and state levels might have also increased individuals’ compliance with social distancing policies. Second, policies should consider socioeconomic inequalities and reinforce specific protective measures in the most vulnerable localities. The poorest are usually at higher risk of infections during pandemics [48] and social distancing showed to be particularly effective in the most vulnerable municipalities. Finally, policies should prioritize regionally coordinated incentives to businesses since local relaxation of social distancing showed insignificant impacts on the local economy.

## Supporting information

S1 Appendix Estimates of the first stage for the dependent variable I, sampled municipalities in São Paulo state, 2020. (DOCX)

S2 Appendix OLS and CF estimates for the dependent variables rate of new cases and rate of new deaths with positive COVID-19 testing, sampled municipalities in São Paulo state, weeks 10 to 27 of 2020. (DOCX)

S3 Appendix OLS and CF estimates for the dependent variables net employment rate and log tax revenue, sampled municipalities in São Paulo state, March to June 2020. (DOCX)

S4 Appendix QLM estimates for interactions between moderator factors and *I*, dependent variables rate of new confirmed cases and rate of new deaths with positive Covid-19 testing, sample of municipalities in São Paulo state, weeks 10 to 27, 2020. (DOCX)

S5 Appendix QLM estimates for interactions between moderator factors and *I*, dependent variable net employment rate, sample of municipalities in São Paulo state, March to June 2020. (DOCX)

S6 Appendix QLM estimates for interactions between moderator factors and I, dependent variable log tax revenue, sample of municipalities in São Paulo state, March to June 2020. (DOCX)
