# Supplementary material for: The short-term impacts of coronavirus quarantine in São Paulo: The health-economy trade-offs
Source: PLoS One. 2021 Feb 17;16(2):e0245011. doi: 10.1371/journal.pone.0245011 (PMC7888633; doi:10.1371/journal.pone.0245011)
Supplement: S1 Appendix — (DOCX) [file pone.0245011.s001.docx]

S1 Appendix – Estimates of the first stage for the dependent variable I, sampled municipalities in São Paulo state, 2020

| Variable | Week | | Month | |
| --- | --- | --- | --- | --- |
| Total precipitation | −0.001 |  | 0.039 | *** |
|  | (0.008) |  | (0.009) |  |
| Mean temperature | −0.004 | * | −0.009 | *** |
|  | (0.002) |  | (0.002) |  |
|  |  |  |  |  |
| Municipalities | 104 | | 104 | |
| Periods | 18 | | 4 | |
| $R^{2}$ (within) | 0.910 | | 0.847 | |

*** p<0.001; ** p<0.01; * p<0.05, + p<0.10. Robust estimates for the standard errors between parentheses.
