# Supplementary material for: The short-term impacts of coronavirus quarantine in São Paulo: The health-economy trade-offs
Source: PLoS One. 2021 Feb 17;16(2):e0245011. doi: 10.1371/journal.pone.0245011 (PMC7888633; doi:10.1371/journal.pone.0245011)
Supplement: S2 Appendix — (DOCX) [file pone.0245011.s002.docx]

S2 Appendix– OLS and CF estimates for the dependent variables rate of new cases and rate of new deaths with positive COVID-19 testing, sampled municipalities in São Paulo state, weeks 10 to 27 of 2020

| Variable | Cases per 1,000 | | | | | | | | Deaths per 1,000 | | | | | | | |
| --- | --- | --- | --- | --- | --- | --- | --- | --- | --- | --- | --- | --- | --- | --- | --- | --- |
|  | Model 1 | | | | Model 2 | | | | Model 1 | | | | Model 2 | | | |
|  | OLS | | CF | | OLS | | CF | | OLS | | CF | | OLS | | CF | |
|  |  |  |  |  |  | + |  |  |  |  |  |  |  | + |  |  |
|  |  |  |  |  |  |  |  |  |  |  |  |  |  |  |  |  |
|  |  |  |  |  |  |  |  |  |  |  |  |  |  |  |  |  |
|  |  |  |  |  |  |  |  |  |  |  |  |  |  |  |  |  |
|  |  |  |  |  |  |  |  |  |  |  |  |  |  |  |  |  |
|  |  |  |  |  |  |  |  |  |  |  |  |  |  |  |  |  |
|  |  |  |  |  |  |  |  |  |  |  |  |  |  |  |  |  |
| Municipalities |  | |  | |  | |  | |  | |  | |  | |  | |
| Weeks |  | |  | |  | |  | |  | |  | |  | |  | |
| (within) |  | |  | |  | |  | |  | |  | |  | |  | |
| Hansen test () |  | |  | |  | |  | |  | |  | |  | |  | |

*** p<0.001; ** p<0.01; * p<0.05, + p<0.10. Robust estimates for the standard errors between parentheses.
