# Supplementary material for: The short-term impacts of coronavirus quarantine in São Paulo: The health-economy trade-offs
Source: PLoS One. 2021 Feb 17;16(2):e0245011. doi: 10.1371/journal.pone.0245011 (PMC7888633; doi:10.1371/journal.pone.0245011)
Supplement: S3 Appendix — (DOCX) [file pone.0245011.s003.docx]

S3 Appendix – OLS and CF estimates for the dependent variables net employment rate and log tax revenue, sampled municipalities in São Paulo state, March to June 2020

| Variable | Net Employment Rate | | | | | | | | Log Tax Revenue | | | | | | | |
| --- | --- | --- | --- | --- | --- | --- | --- | --- | --- | --- | --- | --- | --- | --- | --- | --- |
|  | Model 1 | | | | Model 2 | | | | Model 1 | | | | Model 2 | | | |
|  | OLS | | CF | | OLS | | CF | | OLS | | CF | | OLS | | CF | |
|  |  |  |  |  |  |  |  | + |  |  |  |  |  |  |  |  |
|  |  |  |  |  |  |  |  |  |  |  |  |  |  |  |  |  |
|  |  |  |  |  |  |  |  |  |  |  |  |  |  |  |  | + |
|  |  |  |  |  |  |  |  |  |  |  |  |  |  |  |  |  |
|  |  |  |  |  |  |  |  |  |  |  |  |  |  |  |  |  |
| Municipalities |  | |  | |  | |  | |  | |  | |  | |  | |
| Months |  | |  | |  | |  | |  | |  | |  | |  | |
| (within) |  | |  | |  | |  | |  | |  | |  | |  | |
| Hansen test () |  | |  | |  | |  | |  | |  | |  | |  | |

*** p<0.001; ** p<0.01; * p<0.05, + p<0.10. Robust estimates for the standard errors between parentheses.
