# Supplementary material for: The short-term impacts of coronavirus quarantine in São Paulo: The health-economy trade-offs
Source: PLoS One. 2021 Feb 17;16(2):e0245011. doi: 10.1371/journal.pone.0245011 (PMC7888633; doi:10.1371/journal.pone.0245011)
Supplement: S4 Appendix — (DOCX) [file pone.0245011.s004.docx]

S4 Appendix – QLM estimates for interactions between moderator factors and $I$, dependent variables rate of new confirmed cases and rate of new deaths with positive Covid-19 testing, sample of municipalities in São Paulo state, weeks 10 to 27, 2020

| Variable | Cases per 1,000 | | | | | | | | Deaths per 1,000 | | | | | | | |
| --- | --- | --- | --- | --- | --- | --- | --- | --- | --- | --- | --- | --- | --- | --- | --- | --- |
|  | (1) | | (2) | | (3) | | (4) | | (1) | | (2) | | (3) | | (4) | |
| $I$ | −1.791 |  | 11.566 |  | 39.391 |  | 6.793 |  | −0.287 |  | 1.143 | ^+^ | 4.111 | ^*^ | 0.633 |  |
|  | (6.147) |  | (10.608) |  | (22.562) |  | (8.760) |  | (0.218) |  | (0.626) |  | (1.655) |  | (0.456) |  |
| $I\times65 older$ | 51.182 | ^*^ |  |  |  |  |  |  | 5.481 | ^**^ |  |  |  |  |  |  |
|  | (23.702) |  |  |  |  |  |  |  | (1.915) |  |  |  |  |  |  |  |
| $I\times Poverty$ |  |  | −47.679 | ^*^ |  |  |  |  |  |  | −5.101 | ^**^ |  |  |  |  |
|  |  |  | (22.188) |  |  |  |  |  |  |  | (1.796) |  |  |  |  |  |
| $I\times No water$ |  |  |  |  | −160.597 | ^*^ |  |  |  |  |  |  | −17.142 | ^**^ |  |  |
|  |  |  |  |  | (74.391) |  |  |  |  |  |  |  | (6.053) |  |  |  |
| $I\times Crowding$ |  |  |  |  |  |  | −17.980 | ^*^ |  |  |  |  |  |  | −1.925 | ^**^ |
|  |  |  |  |  |  |  | (8.331) |  |  |  |  |  |  |  | (0.670) |  |
| $\mathbf{wY}$ | 0.486 | ^***^ | 0.486 | ^***^ | 0.486 | ^***^ | 0.484 | ^***^ | 0.391 | ^***^ | 0.391 | ^***^ | 0.391 | ^***^ | 0.391 | ^***^ |
|  | (0.068) |  | (0.069) |  | (0.069) |  | (0.069) |  | (0.041) |  | (0.041) |  | (0.041) |  | (0.041) |  |
|  |  |  |  |  |  |  |  |  |  |  |  |  |  |  |  |  |
| Municipalities | 104 | | 104 | | 104 | | 104 | | 104 | | 104 | | 104 | | 104 | |
| Weeks | 18 | | 18 | | 18 | | 18 | | 18 | | 18 | | 18 | | 18 | |
| $R^{2}$ (within) | 0.600 | | 0.598 | | 0.600 | | 0.600 | | 0.444 | | 0.443 | | 0.443 | | 0.444 | |

*** p<0.001; ** p<0.01; * p<0.05, + p<0.10. Robust estimates for the standard errors between parentheses.
