# Supplementary material for: The short-term impacts of coronavirus quarantine in São Paulo: The health-economy trade-offs
Source: PLoS One. 2021 Feb 17;16(2):e0245011. doi: 10.1371/journal.pone.0245011 (PMC7888633; doi:10.1371/journal.pone.0245011)
Supplement: S5 Appendix — (DOCX) [file pone.0245011.s005.docx]

S5 Appendix – QLM estimates for interactions between moderator factors and $I$, dependent variable net employment rate, sample of municipalities in São Paulo state, March to June 2020

| Variable | Net Employment Rate | | | | | | | | | | | | | |
| --- | --- | --- | --- | --- | --- | --- | --- | --- | --- | --- | --- | --- | --- | --- |
|  | (1) | | (2) | | (3) | | (4) | | (5) | | (6) | | (7) | |
| $I$ | 1.903 |  | −33.665 |  | 33.227 |  | 524.826 |  | 194.713 |  | 84.379 |  | −43.237 |  |
|  | (10.737) |  | (37.541) |  | (22.555) |  | (447.920) |  | (162.103) |  | (61.200) |  | (46.264) |  |
| $I\times Agriculture$ | −290.012 |  |  |  |  |  |  |  |  |  |  |  |  |  |
|  | (180.486) |  |  |  |  |  |  |  |  |  |  |  |  |  |
| $I\times Manufacturing$ |  |  | 297.686 |  |  |  |  |  |  |  |  |  |  |  |
|  |  |  | (254.024) |  |  |  |  |  |  |  |  |  |  |  |
| $I\times Construction$ |  |  |  |  | −511.538 |  |  |  |  |  |  |  |  |  |
|  |  |  |  |  | (443.972) |  |  |  |  |  |  |  |  |  |
| $I\times Retail Trade$ |  |  |  |  |  |  | −2138.301 |  |  |  |  |  |  |  |
|  |  |  |  |  |  |  | (1856.749) |  |  |  |  |  |  |  |
| $I\times Food \& Housing$ |  |  |  |  |  |  |  |  | −2872.941 |  |  |  |  |  |
|  |  |  |  |  |  |  |  |  | (2505.446) |  |  |  |  |  |
| $I\times Social Services$ |  |  |  |  |  |  |  |  |  |  | −342.616 |  |  |  |
|  |  |  |  |  |  |  |  |  |  |  | (277.065) |  |  |  |
| $I\times Others$ |  |  |  |  |  |  |  |  |  |  |  |  | 169.521 |  |
|  |  |  |  |  |  |  |  |  |  |  |  |  | (147.380) |  |
| $\mathbf{wY}$ | −0.018 |  | −0.007 |  | −0.006 |  | −0.007 |  | −0.008 |  | −0.028 |  | −0.010 |  |
|  | (0.063) |  | (0.068) |  | (0.067) |  | (0.067) |  | (0.067) |  | (0.064) |  | (0.067) |  |
|  |  |  |  |  |  |  |  |  |  |  |  |  |  |  |
| Municipalities | 104 | | 104 | | 104 | | 104 | | 104 | | 104 | | 104 | |
| Months | 4 | | 4 | | 4 | | 4 | | 4 | | 4 | | 4 | |
| $R^{2}$ (within) | 0.484 | | 0.399 | | 0.394 | | 0.394 | | 0.394 | | 0.423 | | 0.394 | |

*** p<0.001; ** p<0.01; * p<0.05, + p<0.10. Robust estimates for the standard errors between parentheses.
