# Supplementary material for: The short-term impacts of coronavirus quarantine in São Paulo: The health-economy trade-offs
Source: PLoS One. 2021 Feb 17;16(2):e0245011. doi: 10.1371/journal.pone.0245011 (PMC7888633; doi:10.1371/journal.pone.0245011)
Supplement: S6 Appendix — (DOCX) [file pone.0245011.s006.docx]

S6 Appendix – QLM estimates for interactions between moderator factors and I, dependent variable log tax revenue, sample of municipalities in São Paulo state, March to June 2020

| Variable | Log Tax Revenue | | | | | | | | | | | | | |
| --- | --- | --- | --- | --- | --- | --- | --- | --- | --- | --- | --- | --- | --- | --- |
|  | (1) | | (2) | | (3) | | (4) | | (5) | | (6) | | (7) | |
| $I$ | 1.254 |  | −0.073 |  | 2.457 |  | 21.029 |  | 8.492 |  | 4.286 |  | −0.426 |  |
|  | (2.583) |  | (6.270) |  | (2.912) |  | (61.122) |  | (21.328) |  | (8.057) |  | (6.363) |  |
| $I\times Agriculture$ | −10.807 |  |  |  |  |  |  |  |  |  |  |  |  |  |
|  | (34.320) |  |  |  |  |  |  |  |  |  |  |  |  |  |
| $I\times Manufacturing$ |  |  | 11.217 |  |  |  |  |  |  |  |  |  |  |  |
|  |  |  | (35.501) |  |  |  |  |  |  |  |  |  |  |  |
| $I\times Construction$ |  |  |  |  | −19.255 |  |  |  |  |  |  |  |  |  |
|  |  |  |  |  | (61.210) |  |  |  |  |  |  |  |  |  |
| $I\times Retail Trade$ |  |  |  |  |  |  | −80.834 |  |  |  |  |  |  |  |
|  |  |  |  |  |  |  | (256.692) |  |  |  |  |  |  |  |
| $I\times Food \& Housing$ |  |  |  |  |  |  |  |  | −107.273 |  |  |  |  |  |
|  |  |  |  |  |  |  |  |  | (341.207) |  |  |  |  |  |
| $I\times Social Services$ |  |  |  |  |  |  |  |  |  |  | −12.547 |  |  |  |
|  |  |  |  |  |  |  |  |  |  |  | (39.370) |  |  |  |
| $I\times Others$ |  |  |  |  |  |  |  |  |  |  |  |  | 169.521 |  |
|  |  |  |  |  |  |  |  |  |  |  |  |  | (147.380) |  |
| $\mathbf{wY}$ | 0.092 |  | 0.084 |  | 0.079 |  | 0.087 |  | 0.073 |  | 0.088 |  | 0.084 |  |
|  | (0.060) |  | (0.060) |  | (0.060) |  | (0.061) |  | (0.061) |  | (0.060) |  | (0.060) |  |
|  |  |  |  |  |  |  |  |  |  |  |  |  |  |  |
| Municipalities | 104 | | 104 | | 104 | | 104 | | 104 | | 104 | | 104 | |
| Months | 4 | | 4 | | 4 | | 4 | | 4 | | 4 | | 4 | |
| $R^{2}$ (within) | 0.292 | | 0.288 | | 0.288 | | 0.291 | | 0.299 | | 0.300 | | 0.294 | |

*** p<0.001; ** p<0.01; * p<0.05, + p<0.10. Robust estimates for the standard errors between parentheses.
